# *BnSGS3* Has Differential Effects on the Accumulation of CMV, ORMV and TuMV in Oilseed Rape

**DOI:** 10.3390/v7082815

**Published:** 2015-07-27

**Authors:** Quan Chen, Jie Wang, Mingsheng Hou, Shengyi Liu, Junyan Huang, Li Cai

**Affiliations:** 1 College of Plant Science and Technology of Huazhong Agricultural University, Wuhan 430070, China; E-Mails: chenquan0616@126.com (Q.C.); wantjay@163.com (J.W.); mingshenghou@mail.hzau.edu.cn (M.H.); 2 Xiaozhou Agricultural Service Center in Wanzhou District, Chongqing 404089, China; 3 Oil Crops Research Institute, Chinese Academy of Agricultural Sciences, Key Laboratory of Biology and Genetic Breeding of Oil Crops, the Ministry of Agriculture, Wuhan 430062, China; E-Mails: liusy@oilcrops.cn (S.L.); huangjy@oilcrops.cn (J.H.)

**Keywords:** oilseed rape, virus, *BnSGS3*, virus accumulation, breeding strategy

## Abstract

Virus diseases greatly affect oilseed rape (*Brassica napus*) production. Investigating antiviral genes may lead to the development of disease-resistant varieties of oilseed rape. In this study, we examined the effects of the s*uppressor of gene silencing 3* in *Brassica napus* (*BnSGS3*, a putative antiviral gene) with different genus viruses by constructing *BnSGS3*-overexpressing (*BnSGS3*-Ov) and *BnSGS3*-silenced (*BnSGS3*-Si) oilseed rape (cv. Zhongshuang No. 6) plants. These three viruses are *Oilseed rape mosaic virus* (ORMV), *Turnip mosaic virus* (TuMV) and *Cucumber mosaic virus* (CMV). The native *BnSGS3* expressed in all examined tissues with the highest expression in siliques. All three viruses induced *BnSGS3* expression, but ORMV induced a dramatic increase in the *BnSGS3-*Ov plants, followed by TuMV and CMV. Upon inoculation with three different viruses, transcript abundance of *BnSGS3* gene follows: *BnSGS3*-Ov > non-transgenic plants > *BnSGS3*-Si. The accumulation quantities of ORMV and TuMV exhibited a similar trend. However, CMV accumulation showed an opposite trend where virus accumulations were negatively correlated with *BnSGS3* expression. The results suggest that *BnSGS3* selectively inhibits CMV accumulation but promotes ORMV and TuMV accumulation. *BnSGS3* should be used in different ways (up- and down-regulation) for breeding virus-resistant oilseed rape varieties.

## 1. Introduction

Virus disease, next to white mold caused by *Sclerotinia sclerotiorum*, is one of the most important yield-limiting diseases of oilseed rape production in China [1]. *Turnip mosaic virus* (TuMV, genus *Potyvirus*, family *Potyviridae*), *Cucumber mosaic virus* (CMV, genus *Cucumovirus*, family *Bromoviridae*) and *Oilseed rape mosaic virus* (ORMV, genus *Tobamovirus*, family *Tobamoviridae*) are the main viruses that infect oilseed rape in China [2]. However, to date, no oilseed rape varieties have been bred that are highly resistant to these virus diseases.

RNA silencing, including posttranscriptional gene silencing (PTGS), is an important mechanism of antiviral defense in plants [3,4,5]. In recent years, many studies in the mechanism and relevant antiviral plant genes are reported to search for new methods and resources in disease resistance breeding [4,6,7,8,9,10,11,12]. *Suppressor of gene silencing 3* (*SGS3*) has antiviral activity [4,13,14] and specifically exists in plant functioning as a RNA-binding protein [15]. It has been showed in *Arabidopsis* that *SGS3* is required for PTGS and natural virus resistance [4]. In the cytoplasm of plant cells, SGS3 stabilizes and recruits the viral single-stranded RNAs (ssRNAs) transcripts to RNA-dependent RNA polymerase 6 (RDR6) and RDR6 converts ssRNAs into double-stranded RNAs (dsRNAs) in SGS3/RDR6 bodies [16]. Next, Dicer-like 4 (DCL4) digests the dsRNAs into 21–24 nt primary small interfering RNAs (siRNAs) assisted by double-stranded-RNA-binding protein 4 (DRB4) and part of the dsRNAs are processed into secondary siRNAs by DCL2. These two classes of siRNAs are protected from degradation aided by HUA ENHANCER 1 (HEN1) and then are combined onto the Argonaute 1/2 (AGO1/AGO2) protein and RNA-induced silencing complexes (RISCs) to silence the complementary viral RNAs [3,4,5,9,15,17,18,19]. Furthermore, SGS3 can also function in controlling other kinds of RNA silencing such as those induced by sense transgenes (S-PTGS) [20] or DNA virus-induced gene silencing (VIGS) [21].

CMV, TuMV and *Turnip vein-cleaning virus* (TVCV) have different degrees of inhibitory effects on PTGS [4]. CMV can only partially inhibit PTGS and produces only a little amount of CMV virus RNA in *Arabidopsis* L1 plants, which is significantly lower than that in *sgs*3 mutants. On the contrary, TuMV and TVCV completely inhibit PTGS. As a result, these two viruses accumulated to equally high levels in the *sgs*3 mutants and L1 plants [4]. These results indicate that the PTGS-mediated resistance efficiency on virus infection is related to the inhibitory capability of virus to PTGS.

SGS3 is an essential component of PTGS and closely associated with plant virus resistance. So far, however, the function of *SGS3* in oilseed rape has not been reported. Little is known about the expression of *SGS*3 in oilseed rape or the interaction between major viruses of oilseed rape and SGS3. In this study, we investigated the expression pattern of the *suppressor of gene silencing 3* in *Brassica napus* (designated *BnSGS3*, accession number KP292910) in various oilseed rape tissues. Furthermore, we generated transgenic oilseed rape plants with overexpressed (*BnSGS3*-Ov) and silenced (*BnSGS3*-Si) levels of *BnSGS3*. Using these transgenic plants upon inoculation with three different viruses (ORMV, TuMV and CMV), our results reveal that CMV accumulation was negatively correlated with expression levels of *BnSGS3*, whereas accumulations of both TuMV and ORMV were positively correlated with expression levels of *BnSGS3*, suggesting that *BnSGS3* selectively inhibits CMV accumulation, but promotes TuMV and ORMV accumulation.

## 2. Results

### 2.1. Sequence Comparison and Phylogenetic Analysis of SGS3 Genes in Different Hosts

According to report, SGS3 contains three conservative functional domains: The zinc finger (ZF) domain, the rice gene X and SGS3 (XS) domain and the coiled-coil (CC) domain [16,22,23,24]. The XS domain is a single-stranded RNA-binding domain [22], while the CC domain is related to homodimer formation and the movement of SGS3/RDR6-bodies [16,24]. The N-terminal ZF domain of potato SGS3 was recently shown to control SGS3 location and be the main determinant of the interaction with the viral genome-linked protein (VPg) of *Potato virus* A (PVA) [13]. In this study, a cDNA encoding a homolog of *AtSGS3* was cloned from *Brassica*. *napus* cv. Zhongshuang No.6. The entire open reading frame (ORF) of the cloned cDNA encodes a protein of 607 amino acid residues that exhibit 72.6% identity with AtSGS3 (AF239719). Similar to AtSGS3, the protein sequence contains three conserved domains: ZF domain, XS domain and CC domain, which shared, respectively, 93.3%, 78.3% and 73.3% identity with these domains of AtSGS3. Thus, the cloned cDNA was designated as *BnSGS3* (GenBank accession no. KP292910).

**Figure 1:**
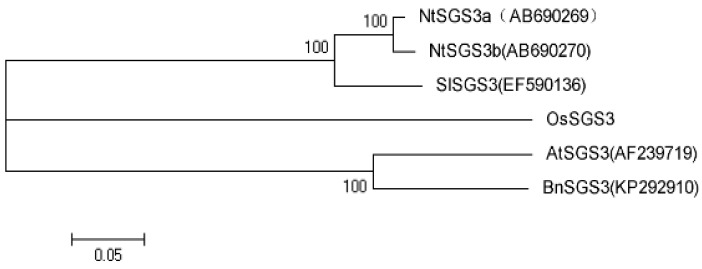
Phylogenetic analysis of the *suppressor of gene silencing 3* in *Brassica napus* (*BnSGS3*) and other published *SGS3* genes involved in plant viral resistance based on the entire open reading frame (ORF) nucleotide sequence. The neighbor-joining tree was generated with the DNAMAN 6.0.4 and Mega 5.0. The significance of the branching order was assessed by bootstrap resampling of 1000 replicates. Values are indicated at the forks. The scale bar corresponds to a 5% difference.

Analysis of phylogenetic relationships would help provide initial insights into degree of structural and functional conservation. Several plant *SGS3* genes, initiated from *Arabidopsis thaliana* (*AtSGS3*) [4], *Solanum lycopersicum* (*SlSGS3*) [25], *Oryza sativa* (*OsSGS3*) [26], and *Nicotiana tabacum* (*NtSGS3*) [27] were implicated in plant viral resistance. Based on alignments, neighbor-joining tree of *SGS3* nucleotide sequences was generated (Figure 1). Results showed that there were three distinct clusters of these *SGS3* genes. The homology was 55.1%–81.0% between *BnSGS3* and other *SGS3* genes. *BnSGS3* was more closely related to *AtSGS3* but distantly to those of *N*. *tabacum*, *S*. *lycopersicum* and *O*. *sativa*. *BnSGS3* and *AtSGS3* belonged to one cluster and they shared the highest homology of 81.0%. The difference between them might relate to the potentially resistance to different viruses.

### 2.2. BnSGS3 Expression in Transgenic and Non-Transgenic Plants of Oilseed Rape

**Figure 2:**
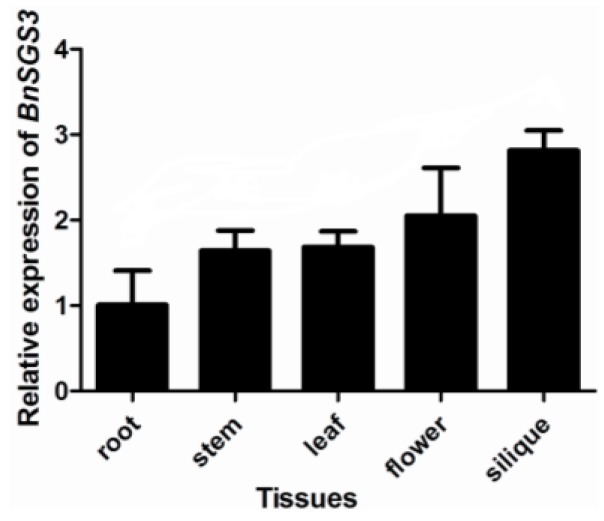
Relative expression levels of *BnSGS3* in different tissues of oilseed rape.

Quantitative RT-PCR (qRT-PCR) was used to detect the expression levels of endogenous *BnSGS3* in different tissues of oilseed rape (*B*. *napus* cv. Zhongshuang No. 6). When the expression level of *BnSGS3* in roots was designated to be 1, the relative expression level in each tissue from high to low followed: Siliques (2.81), flowers (2.05), leaves (1.68), stems (1.64) and roots. The relative expression of *BnSGS3* in roots was only 35% of that in siliques (Figure 2).

To further functionally characterize *BnSGS3*, we produced transgenic oilseed plants. *BnSGS3* overexpression vector (*BnSGS3*-Ov) containing a *Cauliflower mosaic virus* (CaMV) 35S enhancer, double CaMV 35S promoters, an Ω sequence (to improve protein translation), the *BnSGS3* cDNA, and a CaMV Nos terminator within the T-DNA region (Supplementary Figure S1A). *BnSGS3* silenced vector (*BnSGS3*-Si) containing the CaMV 35S promoter, *BnSGS3* sense and antisense repetitive sequences, a Pdk intron and an OCS terminator (Supplementary Figure S1B). These two vectors, each with the pSoup helper plasmid were transformed into the *Agrobacterium tumefaciens* GV3101, which was in turn used to transform oilseed rape cv. Zhongshuang No.6.

**Figure 3:**
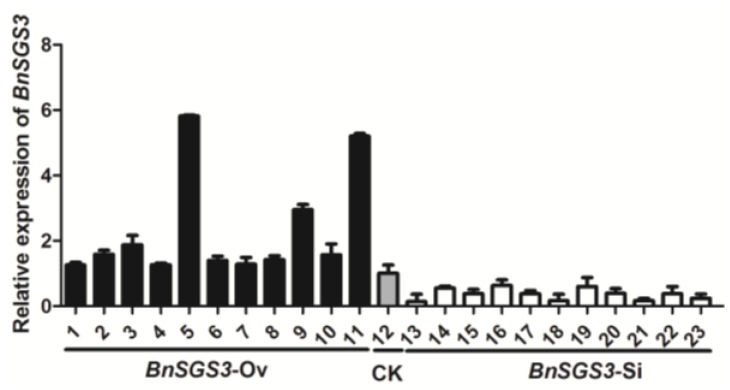
Relative expression levels of *BnSGS3* in single *BnSGS3*-overexpressing (*BnSGS3*-Ov) oilseed rape plants (1–11), non-transgenic oilseed rape plant (12) and single *BnSGS3*-silenced (*BnSGS3*-Si) oilseed rape plants (13–23).

A total of 58 transgenic oilseed rape plants were confirmed by PCR, including 33 *BnSGS3*-Ov and 25 *BnSGS3*-Si transgenic plants. The relative transcript levels of *BnSGS3* in randomly selected 22 plants were determined by qRT-PCR. The results showed that the relative transcript levels of *BnSGS3* among transgenic *BnSGS3*-Ov and *BnSGS3*-Si plants were 144%–581% and 13%–71% of the non-transgenic plants, respectively, indicating that *BnSGS3* expression increased in the *BnSGS3*-Ov plants but was suppressed in the *BnSGS3*-Si plants (Figure 3).

### 2.3. Dynamics of BnSGS3 Expression in T*_0_* Generation Transgenic and Non-Transgenic Plants of Oilseed Rape after Viral Infection

**Figure 4:**
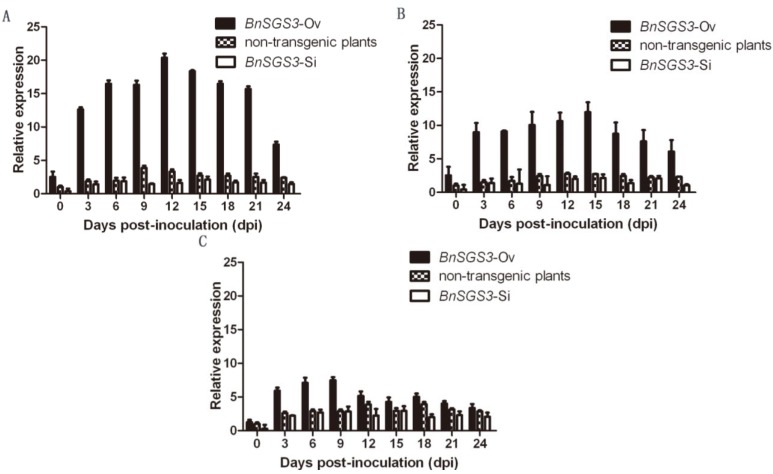
Dynamic expression of *BnSGS3* induced by (**A**) *Oilseed rape mosaic virus* (ORMV); (**B**) *Turnip mosaic virus* (TuMV) and (**C**) *Cucumber mosaic virus* (CMV) in the transgenic and non-transgenic plants. The expression level of *BnSGS3* in non-transgenic plants uninoculated was designated to be 1.

To test the effects of *BnSGS3* expression and the accumulation of viruses, each 50 ng of purified ORMV (*Tobamovirus*, subgroup III strain), TuMV (*Potyvirus*, MB cluster strain) and CMV (*Cucumovirus*, subgroup I strain) viruses were, respectively, inoculated onto five T_0_ generation transgenic and non-transgenic plants at the four-leaf-stage. Analyses of the changes in expression of *BnSGS3* after inoculation showed that all three viruses induced the expression of *BnSGS3* in both transgenic and non-transgenic oilseed rape. Generally, the expression of *BnSGS3* first increased and then dropped (Figure 4). In all three viruses’ inoculation treatments, transcript levels of *BnSGS3* in overexpressing T_0_ generation plants were significantly greater than those in the non-transgenic and *BnSGS3* silenced plants while expression in the non-transgenic plants was slightly higher than that in the silenced plants. In *BnSGS3*-overexpressing plants upon virus inoculation, three viruses showed different induction on *BnSGS3* expression, most efficiently by ORMV followed by TuMV and CMV, while the inductive effect of CMV on *BnSGS3* expression was slightly stronger than those by ORMV and TuMV in *BnSGS3*-Si transgenic and non-transgenic plants. These results suggest that in both transgenic and non-transgenic plants of oilseed rape, all three viruses ORMV, TuMV and CMV were able to induce expression of *BnSGS3*, but induction capability is different.

### 2.4. Effect of BnSGS3 on the Accumulation of ORMV, TuMV and CMV

A diluted concentration gradient of the plasmid containing viral *CP* gene fragments was subjected to qRT-PCR amplification. The Ct values of a gradient of viral plasmid concentrations were used for the Y-axis, and logarithm (log10) values of the copy number (copies/μL) were used for the X-axis to construct a standard curve (Supplementary Figure S2). The results showed that the amplification efficiency (E) of ORMV, TuMV, and CMV viral plasmids was 86.2%, 88.7% and 84.9%, respectively, and R^2^ was greater than 0.99, indicating that there was a good linear relationship between Ct values and the logarithm of the copy numbers and the standard curves were appropriate for calculation of virus quantity.

**Figure 5:**
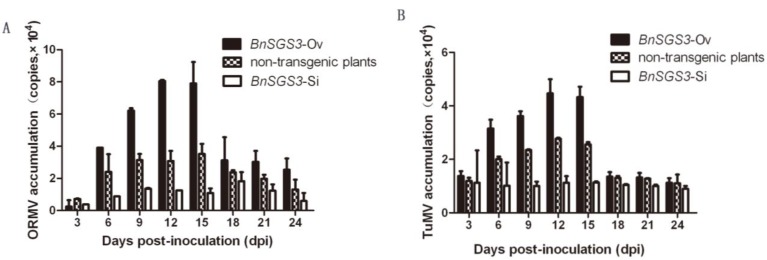

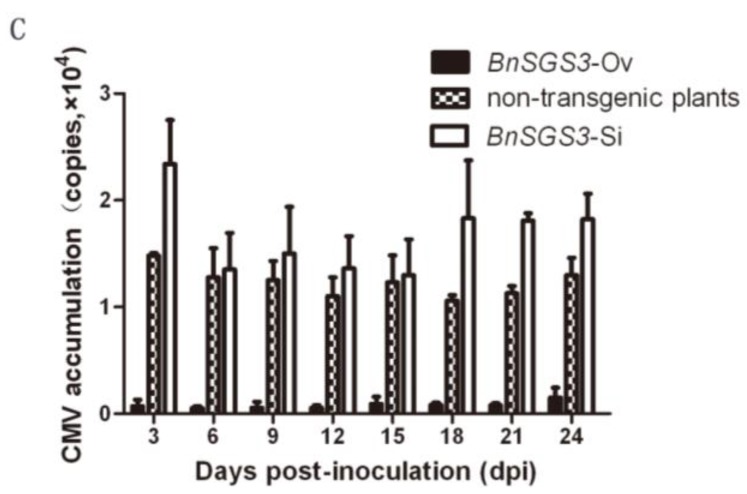
Accumulation of ORMV (**A**); TuMV (**B**) and CMV (**C**) in the transgenic and non-transgenic oilseed rape plants.

To investigate whether the *BnSGS3* expression effect the viral accumulation, five T_0_ generation transgenic and non-transgenic plants were inoculated with 50 ng of the purified ORMV, TuMV and CMV particles at the four-leaf-stage. The viral accumulation was measured in 0–24 days of post-inoculation (dpi) at intervals of three days. After inoculation of *BnSGS3*-Ov, oilseed rape plants with ORMV and TuMV, virus levels first increased and then decreased, reaching peak values of 8.05 × 10^4^ copies and 4.47 × 10^4^ copies at 12 dpi, respectively. In non-transgenic oilseed rape, the virus levels initially increased, reaching peak values of 3.52 × 10^4^ copies and 2.77 × 10^4^ copies at 15 and 12 dpi, respectively, followed by a decrease. However, in *BnSGS3*-Si oilseed rape inoculated with ORMV and TuMV, the virus levels reached peak values of 1.82 × 10^4^ copies and 1.14 × 10^4^ copies at 18 and 15 dpi, respectively. These results demonstrate that the quantities of ORMV and TuMV exhibited a trend: *BnSGS3*-Ov plants > non-transgenic plants > *BnSGS3*-Si plants (Figure 5A,B), consistent with *BnSGS3* expression levels in these type plants.

The replication rate of CMV in *BnSGS3*-Ov plants was small and its accumulation levels were low, reaching a peak of 1.50 × 10^3^ copies at 24 dpi, and the amplitude of variation of qRT-PCR throughout the entire test time course was small. In inoculated non-transgenic and *BnSGS3*-Si plants, increase of CMV levels was relatively quick, reaching a peak of 1.48 × 10^4^ copies and 2.34 × 10^4^ copies at 3 dpi, respectively. The levels fluctuated a bit, but the changes were small (Figure 5C). In non-transgenic oilseed rape, CMV levels were much higher with 1.5 × 10^4^ copies than that of ORMV at 3 dpi and afterwards virus quantities were ORMV > TuMV > CMV. The general trend of CMV accumulation was the *BnSGS3*-Si > non-transgenic plants > *BnSGS3*-Ov, in contrary to both the induction expression trend of *BnSGS3* and the accumulation trends of ORMV and TuMV. Furthermore, virus accumulation of ORMV and TuMV in the *BnSGS3*-Ov are drastically higher than that of CMV at 6 dpi and afterwards.

### 2.5. Correlation between BnSGS3 Expression and Virus Accumulation

**Figure 6:**
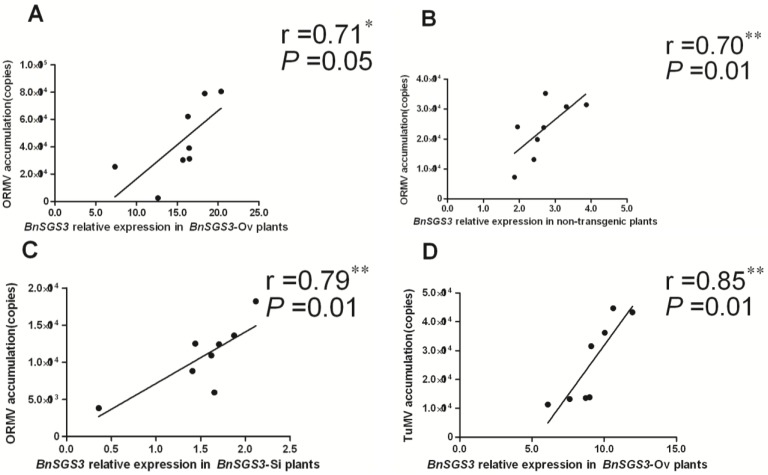

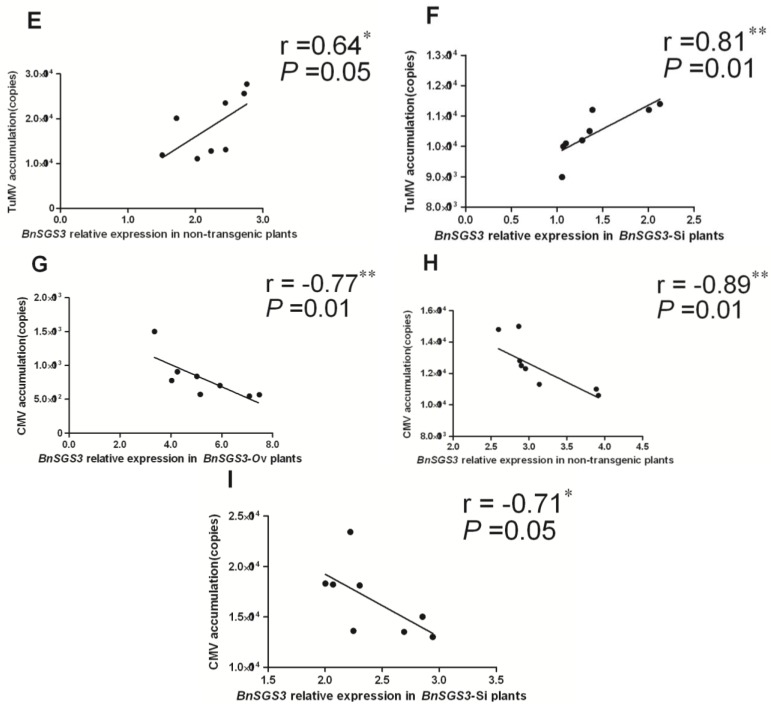
Relationship between the accumulation level of ORMV (**A**–**C**), TuMV (**D**–**F**) or CMV (**G**–**I**) and the expression quantity of *BnSGS3* in *BnSGS3*-Ov (**A**,**D**,**G**), non-transgenic (**B**,**E**,**H**) and *BnSGS3-*Si (**C**,**F**,**I**) plants.

SPSS17.0 statistical software was used to analyze the relationship between virus accumulation and *BnSGS3* expression levels in transgenic and non-transgenic oilseed rape. There was a positive and significant (*p* < 0.01 or 0.05) linear correlation between the accumulation of ORMV and TuMV (in transgenic and non-transgenic plants) and *BnSGS3* expression (Figure 6A–F). However, the relationship between CMV accumulation and *BnSGS3* expression in transgenic and non-transgenic oilseed rape exhibited a negative and significant (*p* < 0.01 or 0.05) linear correlation (Figure 6G–I). These results indicated that the expression of *BnSGS3* promoted the accumulation of ORMV and TuMV but suppressed CMV accumulation.

## 3. Discussion

In this study, we first reported the oilseed rape *SGS3* gene (*BnSGS3*) and determined the relationship between the *BnSGS3* expression and the virus accumulation of ORMV, TuMV and CMV, which are the main viruses that infect oilseed rape. For ORMV and TuMV, after inoculation of transgenic and non-transgenic oilseed rape plants, differences in the induced expression of *BnSGS3* were significant. The higher the level of *BnSGS3* expression was, the higher the corresponding accumulation of ORMV and TuMV was. Furthermore, in *BnSGS3*-Si plants, the expression of *BnSGS3* was low, and the virus accumulation was also low. Correlation analysis showed that there was a positive linear correlation between the two (Figure 6A–F). Similar phenomenon was observed in PVA and *Soybean mosaic virus* (SMV). A study of the interaction between PVA VPg protein and potato SGS3 (StSGS3) revealed that the accumulation of PVA in *StSGS3-*overexpressing plants was higher than in *StSGS3-*silenced plants [13]. Likewise, there is a lower level of SMV strain G7 RNA in the *Glycine max SGS3* (*GmSGS3*)-silenced soybean than non-silenced plants. The resulting severity of lethal systemic hypersensitive response (LSHR) in *GmSGS3*-silenced soybean was also alleviated [14].

In our research, we found that the accumulation of ORMV and TuMV increased accompanied by the up-regulated expression of *BnSGS3*, suggesting that these two viruses can successfully inhibit RNA silencing in plants [28]. So far, many viruses have been reported to have this function [29,30,31,32,33], which may be relevant to the virus suppressors of RNA silencing (VSRs). Among these VSRs, HC-Pro protein encoded by potyviruses (TuMV also encodes this HC-Pro) is a highly effective VSR that not only inhibits silencing but can also reverse an already established RNA silencing [34,35]. The expression of HC-Pro does not inhibit the production of a systemic silencing signal but prevents the accumulation of the siRNAs [36]. In *Tobamovirus* (ORMV is included in this category), both P126 of *Tobacco mosaic virus* (TMV) [5] and 130k of *Tomato mosaic virus* (ToMV) [37] can function as VSRs to prevent RNA silencing. Therefore, *BnSGS3* may have a role in ORMV and TuMV accumulation effect through closely linked to VSRs. Since SGS3 stabilizes the viral ssRNA transcripts toRDR6 [16], it is possible that these two viruses’ VSRs directly or indirectly interact with BnSGS3 and recruit BnSGS3 to protect viral RNA from degradation [13,15,21,25,26,38]. Alternatively, the VSRs prevent BnSGS3 from binding and accessing their common substrate RNAs, which lead to inhibition of RNA silencing [15]. Another possibility is that the accumulation differences may be induced by interference in the amplification step of RNA silencing via a yet-unidentified mechanism [13]. Many studies have reported that sense-mediated RNA silencing may interlink with other RNA quality control systems [11,16,39]. Therefore, it is possible that TuMV and ORMV encoding VSRs interact with BnSGS3 in both RNA silencing and other interlinked RNA quality control systems, which also need BnSGS3 to inhibit RNA silencing.

By contrast, the accumulation level of CMV in different oilseed rape plants was differed from ORMV and TuMV. The peak value of CMV accumulation in various plants exhibited the trend *BnSGS3*-Si (2.34 × 10^4^) > non-transgenic oilseed rape (1.48 × 10^4^) > *BnSGS3*-Ov (1.50 × 10^3^; Figure 5C). The opposite trend was observed for CMV-induced *BnSGS3* expression. Therefore, the higher the level of *BnSGS3* expression was, the lower the level of CMV was. Correlation analysis revealed a negative linear correlation between the two factors (Figure 6G–I). Previous studies also showed 5-fold over accumulation of CMV RNA in *sgs3* mutant [4] and CMV 2b deficient mutant failed to infect wild-type *Arabidopsis* but was highly virulent in *sgs3* mutant [40], which suggested that the CMV 2b protein did not target the silencing mechanism in the same way with HC-Pro [30,31]. During infection, CMV 2b directly interacted with AGO1 and specifically inhibited its slicing activity [41,42,43,44]. Since AGO1 was dispensable for RDR-dependent production of CMV secondary siRNAs, which revealed that the antiviral pathway might differ from the *trans*-acting siRNAs (tasiRNAs) pathway [5]. Thinking of our results, excepting BnSGS3, there may also be one or more RNA silencing genes, such as RDR6 or AGO1, essential for silencing amplification, contribute to the biosynthesis of CMV secondary siRNAs. In a word, the CMV accumulation effect suggests that there is a work way differs from TuMV and ORMV, and the CMV accumulation strategy has some intimate relationships with BnSGS3, RDR6 and AGO1, but the exact mechanism needs to be further studied.

In summary, the effects of *BnSGS3* expression on the accumulation of ORMV, TuMV and CMV in oilseed rape are different. *BnSGS3* may promote ORMV and TuMV accumulation, but inhibit CMV accumulation, suggesting different strategies for use of *SGS3* (up- and down-regulation) for breeding resistant oilseed rape against various viral diseases.

## 4. Materials and Methods

### 4.1. Plant Materials

Oilseed rape (*Brassica napus*) cv. Zhongshuang No. 6, which is low susceptible to the viruses, was used for virus inoculation and for the analysis of *BnSGS3* expression in this study. Oilseed rape seedlings were grown in the greenhouse with a light/dark cycle of 14/10 h at 22 °C (D)–24 °C (L). The humidity in the greenhouse was 50%–70%.

### 4.2. Sequence Analysis of BnSGS3 and Vector Construction

The primers used in this study are shown in Table 1. For *BnSGS3* cDNA amplification, total RNA of *B. napus* leaves was extracted by using total RNA kit I (OMEGA, Norcross, GA, USA), and 1 μg RNA was reverse transcribed to cDNA (RevertAid First Strand cDNA Synthesis Kit, Thermo, Waltham, MA, USA). A 1974 bp cDNA containing the entire open reading frame (ORF) of *BnSGS3* was PCR amplified from its cDNA with primers Bn-F1 and Bn-R1. Software DNAMAN6.0.4 and Mega5.0 were used for sequence alignments and generated a neighbor-joining tree.

To construct a vector for constitutive expression of *BnSGS3*, the vector pG4A was generated by inserting a fragment from pTΩ4A containing a CaMV 35S enhancer, double CaMV 35S promoters, and a CaMV Nos terminator into the *Kpn*I-*Not*I site of pGreen0229 containing a *bar* gene within the T-DNA region for selecting transgenic plants. A 1848 bp fragment containing the entire ORF segment of *BnSGS3* was PCR amplified from its cDNA clone with primers Bn-F2 and Bn-R2, and inserted into the multiple cloning sites (*Xho*I) of pG4A, creating a *BnSGS3* overexpressing vector *BnSGS3*-Ov (Figure S1A).

Vectors pGreen0229 and pKANNIBAL were used as primary and intermediate vectors to construct the *BnSGS3* silenced vector. A 404-bp *BnSGS3* sense (amplified by BnRI-C and BnRI-KC, containing *Xho*I and *EcoR*I sites) and antisense (amplified by BnRI-A and BnRI-E, containing *Xba*I and *Hind*III sites) fragments were inserted into both ends of pKANNIBAL Pdk introns. Then, *Not* I was used to extract the product containing the CaMV 35S promoter, *BnSGS3* sense and antisense repetitive sequence, Pdk intron and OCS terminator from pKANNIBAL, and inserted into the *Not* I site of pGreen0229, creating a *BnSGS3* silenced vector *BnSGS3*-Si (Figure S1B).

All the inserted sequences were confirmed by enzyme digestion and sequencing. The resulting *BnSGS3*-Ov and *BnSGS3*-Si vectors together with the pSoup helper plasmid [45,46] were transformed into the *Agrobacterium tumefaciens* GV3101 by electroporation for plant transformation.

**Table 1:** List of primers.

Primer	Sequence (5′-3′)	Tm (°C)	Product (bp)
*BnSGS3* cDNA amplification			
Bn-F1	TGAGGTTCTGGACAGGGATC	55	1974
Bn-R1	GCCGTCTTACTGAAAATGGA		
Vector construction			
Bn-F2	GTACTCGAGGTTTGCTCTCTGTTTGGTT	55	1848
Bn-R2	CGTCTCGAGGCTAGTAGTCTTCTGTGTC		
BnRI-C	TATACTCGAGCAGTGGAAGGGTTTGGGTG	54	404
BnRI-KC	GCCCGAATTCTCCCTACTTGACAGTGTTG		
BnRI-A	CGAATCTAGACAGTGGAAGGGTTTGGGTG	54	404
BnRI-E	GCCGAAGCTTTCCCTACTTGACAGTGTTG		
Detection of expression quantity			
Bn-F	TGGAAGGGTTTGGGTGAGGAG	62	181
Bn-R	GTGGACCATAGGAGTGGCGTG		
Actin-F	CTGGAATTGCTGACCGTATGAG	62	145
Actin-R	ATCTGTTGGAAAGTGCTGAGGG		
Bar-F	TGCCAGAAACCCACGTCAT	55	485
Bar-R	CTGCACCATCGTCAACCACTA		
Detection of virus accumulation			
CMV-F	CCTCACCGGTACTGGTTTATC	62	107
CMV-R	CTTTCGCATGTCGCCAATATC		
TuMV-F	GGAAGTAAACGCTGGAACCT	62	96
TuMV-R	GCCACTCTTTGCTCGTATCT		
ORMV-F	CTGTGGCCATTAGGAGTCAA	62	108
ORMV-R	GCGCAGTAGTCCAAGGTAATA		

### 4.3. Production of BnSGS3-Ov and BnSGS3-Si *Brassica napus* Transformants

*Brassica napus* cv. Zhongshuang No. 6 was transformed using an improved transformation method based on the method of Liu *et al.* [47]. Rapeseeds were soaked in 5% sodium hypochlorite for 3–5 min and plated onto M0 medium (1/2MS). After 6 days of cultivation in the dark, the hypocotyledonary axis of each seedling was co-cultured with *A. tumefaciens* liquid culture containing the *BnSGS3*-Ov or *BnSGS3*-Si plasmids for 30 min. The hypocotyledonary axes were then transferred to M1 medium (MS + 30 g/L sucrose + 18 g/L mannitol + 1 mg/L 2,4-D + 0.3 mg/L kinetin + 100 μM acetosyringone + 8.5 g/L agarose, pH 5.8) and cultured in the dark at 24 °C for 2–3 days, followed by culture on M2 medium (MS + 30 g/L sucrose + 18 g/L mannitol + 1 mg/L 2,4-D + 0.3 mg/L kinetin + 20 mg/L AgNO_3_ + 8.5 g/L agarose + 25 mg/L kanamycin + 250 mg/L carbenicillin, pH 5.8) at 24 °C for 3 weeks. Hypocotyl callus was then transferred to M3 medium (MS + 10 g/L glucose + 0.25 g/L xylose + 0.6 g/L MES hydrate + 2 mg/L zeatin + 0.1 mg/L indole-3-acetic acid + 8.5 g/L agarose + 25 mg/L kanamycin + 250 mg/L carbenicillin, pH 5.8) and subcultured once every 3 weeks until the emergence of green shoots, which were cultured on M4 medium (MS + 10 g/L sucrose + 10 g/L agar, pH 5.8) for 3–4 weeks for rooting.

Primers Bar-F and Bar-R (see Table 1) were utilized to identify transgenic plants. Each transgenic plant was used to produce at least three plants, which were used as materials in independent repeated trials. The plants were transplanted into pots at the 3-leaf-stage and grown in the greenhouse as described above.

### 4.4. Virus Purification and Inoculation

ORMV (*Tobamovirus*, subgroup III strain), TuMV (*Potyvirus*, MB cluster strain) and CMV (*Cucumovirus*, subgroup I strain) were originally isolated from systemically infected oilseed rape (*B. napus* L. var. *oleifera*) in Hubei Province, China. Chinese cabbage (*Brassica rapa*) was used to grow and multiply these viruses by mechanical inoculation [48]. Three to four weeks post-inoculation, young emerging leaves of infected plants were harvested. Virus purification was performed as described by Aguilar *et al.* [49]. About 50 ng of the purified ORMV, TuMV or CMV particles were mechanically inoculated [48] onto the leaves of transgenic T_0_ generation and non-transgenic oilseed rape plants at the 4-leaf-stage. Each virus was inoculated onto five plants of each type, with two independent biological replications. Thirty plants were inoculated per *BnSGS3*-Ov, *BnSGS3*-Si and non-transgenic oilseed rape plants.

### 4.5. Sample Collection and RNA Extraction

To detect differences in *BnSGS3* expression in different tissues of oilseed rape and in each transgenic T_0_ generation plant, the tender roots, stems, leaves, flowers and siliques of oilseed rape and the tender leaves of each transgenic plant were collected at the same time. After inoculation with one of three types of virus, 0, 3, 6, 9, 12, 15, 18, 21 and 24 days post-inoculation (dpi, as well as the uninoculated control), tender leaves from the upper parts of plants were used to detect virus-induced *BnSGS*3 expression and the amount of virus accumulation. Total RNA was extracted from the above samples (E.Z.N.A. Total RNA Kit I, OMEGA, Norcross, GA, USA), and 1 μg RNA was reverse transcribed to cDNA (RevertAid First Strand cDNA Synthesis Kit, Thermo, Waltham, MA, USA).

### 4.6. Quantitative RT-PCR

Quantitative RT-PCR (qRT-PCR) was performed to detect the expression levels of *BnSGS3* in different tissues of non-transgenic oilseed rape plants, single transgenic plants and the virus-inoculated plants. Primers Actin-F and Actin-R were used to amplify the reference β*-Actin* gene (AF111812), and primers Bn-F and Bn-R were used to amplify *BnSGS3* (all primers are listed in Table 1). The relative transcript levels of *BnSGS3* 2^−ΔΔCt^ were obtained by calculating ΔCt values [∆Ct = Ct(*BnSGS3*)-Ct(*Actin*)].

qRT-PCR was also used to detect the accumulation of three viruses (by copy number) in the samples. Primers ORMV-F/R, CMV-F/R and TuMV-F/R were used to amplify *CP* gene fragments of the three viruses via PCR. The PCR products were ligated to pMD-18T, which was transformed into *E. coli* DH5α, and plasmid DNA was subsequently extracted (TIANprep Mini Plasmid Kit, TIANGEN). The molecular weights of the ORMV, TuMV and CMV plasmids were 1,730,750.4 Da, 1,730,135 Da and 1,723,338.6 Da, respectively. The copy number of 1 μL plasmids was calculated according to Avogadro’s formula “6.022 × 10^23^ molecules/moL”, and the plasmids were diluted to 1.0 × 10^9^–1.0 × 10^3^ copies/μL concentration gradient, with three repeats per gradient, which were simultaneously subjected to qRT-PCR [50,51]. The copy numbers of the three viruses in different samples were automatically calculated based on the sample Ct values and the standard curve.

Two independent biological replicates and three technical replicates were carried out for the inoculation experiment for each virus, taking the average value of two sets of data. qRT-PCR was performed on the BioRad CFX96 Real-Time System (C1000 Thermal Cycler). The reaction system included 10 μL SYBR Green PCR Master Mix, 0.5 μL of each upstream and downstream primer (10 μmol/L), 1 μL cDNA and 8 μL ddH_2_O. The reaction conditions were as follows: 95 °C for 3 min, 95 °C for 10 s, 62 °C for 30 s, 72 °C for 15 s (40 cycles), and 65 °C for 5 s.

### 4.7. Correlation Analysis of Virus Accumulation *vs.* BnSGS3 Expression

SPSS17.0 statistical software and Pearson’s two-sided test were used to analyze whether there was a correlation between the accumulation of three types of viruses and *BnSGS3* expression levels, as well as the level of significance.

## Supplementary Files

Supplementary File 1
