# Supplementary material for: BnSGS3 Has Differential Effects on the Accumulation of CMV, ORMV and TuMV in Oilseed Rape
Source: Viruses. 2015 Jul 27;7(8):4169–85. doi: 10.3390/v7082815 (PMC4576176; doi:10.3390/v7082815)
Supplement: Supplementary File 1 [file viruses-07-02815-s001.pdf]

Supplementary Information

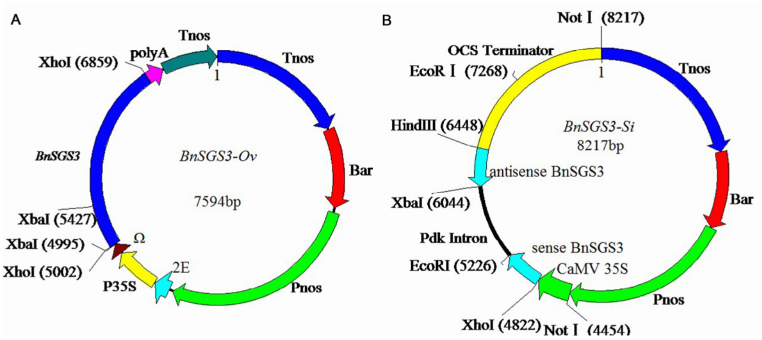

Figure S1. The schematic diagram of the *suppressor of gene silencing 3* in *Brassica napus* (*BnSGS3*)-overexpressing vector (*BnSGS3*-Ov) (**A**) and *BnSGS3*-silenced vector (*BnSGS3*-Si) (**B**). (**A**) *BnSGS3*-Ov containing the *Cauliflower mosaic virus* (CaMV) 35S enhancer, double CaMV 35S promoter, Ω sequence, *BnSGS3* cDNA, and the CaMV Nos terminator; (**B**) *BnSGS3*-Si containing CaMV 35S promoter, *BnSGS3* sense and antisense repetitive sequence, Pdk intron and OCS terminator.

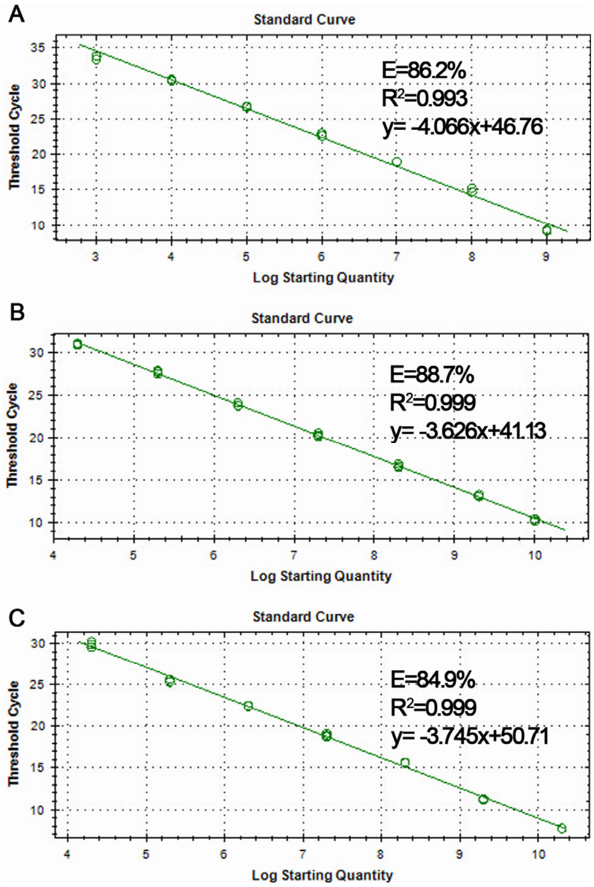

Figure S2. Standard curves for three viruses' amplification. (**A**) Standard curve for *Oilseed rape mosaic virus* (ORMV) viral plasmid; (**B**) Standard curve for *Turnip mosaic virus* (TuMV) viral plasmid; (**C**) Standard curve for *Cucumber mosaic virus* (CMV) viral plasmid.

© 2015 by the authors; licensee MDPI, Basel, Switzerland. This article is an open access article distributed under the terms and conditions of the Creative Commons Attribution license (http://creativecommons.org/licenses/by/4.0/).
